# Heart transplant outcomes in restrictive cardiomyopathy: UNOS registry analysis of the last three decades

**DOI:** 10.1016/j.jhlto.2023.100031

**Published:** 2023-12-05

**Authors:** Daniel J. Miklin, Eugene C. DePasquale

**Affiliations:** a North Shore University Hospital, Northwell Health, Manhasset, New York; b University of Southern California School of Medicine, Los Angeles, California

**Keywords:** heart transplantation, UNOS, restrictive cardiomyopathy, transplant outcomes, heart failure

## Abstract

**Background:**

Restrictive cardiomyopathy (RCM) comprises diverse etiologies with overall poor prognosis. Emerging therapies have significantly impacted some of these entities. However, these therapies may have limited impact in the end-stages and have only recently become available. We sought to assess outcomes before and after transplant in the RCM population stratified into 3 distinct time periods from the recent era.

**Methods:**

Retrospective analysis of the United Network of Organ Sharing registry (*n* = 62,709) for all patients transplanted between 1987 and March 1, 2022, were stratified by RCM status with 1157 patients with RCM. Populations were grouped temporally into *classic* (1987-2000), *contemporary* (2000-2013), and *current* (2014-2022) eras. Multiorgan and repeat transplants were excluded from the analysis. Baseline demographics, listing status, hemodynamics, donor information, and life support methods were compared using Kruskal-Wallis and Pearson’s tests. Longitudinal survival was assessed via Kaplan-Meier survival analysis. Univariate and multivariate analyses using Cox modeling and competing outcomes analyses were performed.

**Results:**

RCM patients were older, female, with older donors and longer ischemic times (*p* < 0.001). There were no significant differences in overall survival compared to the non-RCM population, however, with increased transplant rates. Amyloidosis and chemotherapy/radiation portend the worst prognosis but have shorter waitlist times and up-trending survival in the *current* era.

**Conclusions:**

RCM represents a small but significant population of those requiring heart transplant. RCM transplant outcomes appear to be improving across all subsets with shorter wait times and better survival. Early recognition is important to help mitigate adverse outcomes.

## Background

Restrictive cardiomyopathy (RCM) is a rare and often under-reported disorder that includes infiltrative, hereditary/storage disorders, iatrogenic, and idiopathic cardiomyopathies associated with myocardial stiffness and impaired ventricular filling. Common etiologies include amyloidosis, storage disorders (Anderson-Fabry disease, Pompe disease, hemochromatosis) mutations in cardiac proteins (sarcomeric, cytoskeletal, envelope, titin), radiation-induced, medication-induced, and undifferentiated RCM.^1^, ^2^, ^3^ Appropriate diagnosis can remain elusive and present late in the disease course, as such the prognosis of RCM regardless of cause is very poor and many patients progress to end-stage heart failure.^4^, ^5^ Emerging therapies have impacted some types of RCM, but ultimately end-stage RCM can only be cured through cardiac transplantation.

Prior analyses on RCM have demonstrated varied survival of RCM compared to non-RCM heart transplant recipients. In general, RCM patients tend to have worse outcomes as many of them are present late in their disease course and have limited support options due to the physiology of their disease. Further the etiology of their disease may be progressive, irreversible, and include other organ systems. However, since the changes in United Network of Organ Sharing (UNOS) listing criteria in October 2018, the wait times and transplant rates of RCM patients appear to be improving.^6^ However, data are limited with RCM subtypes. For example, amyloidosis, a progressive and traditionally poor-prognosis subset of RCM patients, appears to have improved outcomes in the current era as compared to prior.^7^ Whether is this due to changes in transplant listing criteria or an improvement in disease-specific therapies is unclear, but it merits further investigation. Similarly, diagnostic tools for familial RCM and improved cancer therapies have likely led to an increased prevalence in RCM-related transplantation.^8^ Previously, UNOS registry analysis of RCM patients from 1987 to 2010 demonstrated increased mortality for radiation-induced and amyloidosis RCM.^9^ As transplantation management and therapeutic options for specific forms of RCM have advanced since these analyses, we sought to reassess RCM outcomes following transplantation in a contemporary population.

## Methods

### Patient identification and selection

The UNOS maintains a registry of clinical and demographic data of organ transplants in the United States. We queried the UNOS database for Thoracic registry patients ≥18 years of age who underwent heart transplantation between March 1, 1987, and March 1, 2022. Study population included only single heart transplants (*n* = 62,709). Redo transplants were excluded from the analysis and multiorgan transplants were separated to limit any confounding factors.

Baseline demographic, clinical, hemodynamic, donor, and listing results were obtained from the registry for all patients, and data were divided into those with a diagnosis of RCM (*n* = 1,157) or non-RCM. Populations were grouped temporally into *classic* (1987-2000), *contemporary* (2000-2013), and *current* (2014-2022) eras. The RCM cohort was subdivided into idiopathic, amyloid, radiation/chemotherapy, and other. Additionally, the UNOS dataset does not delineate amyloid subtype (light chain vs transthyretin) or type of chemotherapy/radiation administered.

### Statistical analysis

Patient demographics, donor information, and clinical characteristics were compared first using the RCM and non-RCM groups, then again between the 4 RCM subgroups. Continuous variables were analyzed by mean and standard deviation. Categorical variables were reported as proportions or percentages. The Kruskal-Wallis test was used for continuous variables and Pearson’s tests for categorical variables.

Longitudinal survival was assessed using the Kaplan-Meier method and crude mortality was compared between groups using the log-rank test.Follow-up time was censored at 12 years (144 months). Univariate and multivariate Cox proportional hazard regression models were used to estimate mortality with adjusted and unadjusted pretransplant and post-transplant risk factors and characteristics. Competing outcomes analysis was assessed at 3-month, 6-month, and 12-month intervals.

Pretransplant variables included recipient age, recipient gender, ethnicity, UNOS listing status, RCM subtype, diabetes, cardiac surgery history, life support presence and type, presence of ventricular assist device, ventilator time, days on the waitlist, serum creatinine, smoking history, hemodialysis, panel reactive antibodies (PRA), and cardiac vs noncardiac death. Significance level was reported as *p* ≤ 0.05.

## Results

### Incidence

Over the 35-year study period, 61,552 non-RCM patients underwent single organ first time heart transplantation as compared to 1,157 (1.8%) individuals with RCM (Table 1). For non-RCM patients, the most common indications for transplant were dilated cardiomyopathy (47.7%) and ischemic cardiomyopathy (42.9%) as compared to hypertrophic cardiomyopathy (2%) and other (7.4%). In the RCM cohort, amyloid (49.4%) and idiopathic (31.2%) comprised the majority of transplants compared to radiation/chemotherapy (8.4%) and other (11%) etiologies. Temporal analysis showed significant changes in the percentage of non-RCM vs RCM patients listed per era: *classic* (1987-2000) (33.7% vs 14%), *contemporary* (2001-2013) (34% vs 35.1%), and *current* (2014-2022) (32.2%. vs 50.9%) *p* < 0.001 (Table S1).

**Table 1:** Baseline Characteristics and Clinical Data for RCM and Non-RCM Heart-Only Transplant Recipients

Variable	Non-RCM	RCM	*p*-value
N	61,552 (98.2%)	1,157 (1.8%)	
Recipient age, mean (SD) years	**52.689 (11.939)**	**54.689 (13.258)**	**<0.001**
Recipient sex			
Female	**14,711 (23.9%)**	**401 (34.7%)**	**<0.001**
Male	46,841 (76.1%)	756 (65.3%)	
Ethnicity			
White	**44,820 (72.8%)**	**811 (70.1%)**	**0.008**
Black	10,480 (17.0%)	243 (21.0%)	
Hispanic	4,241 (6.9%)	66 (5.7%)	
Asian	1,470 (2.4%)	31 (2.7%)	
American Indian/Alaska Native	207 (0.3%)	1 (0.1%)	
Native Hawaiian/Other Pacific Islander	141 (0.2%)	4 (0.3%)	
Multiracial	168 (0.3%)	1 (0.1%)	
Unknown	25 (0.0%)	0 (0.0%)	
Cardiomyopathy type			
Ischemic	**26,390 (42.9%)**	**0 (0.0%)**	**<0.001**
Restrictive	0 (0.0%)	1,157 (100.0%)	
Hypertrophic	1,229 (2.0%)	0 (0.0%)	
Dilated	29,341 (47.7%)	0 (0.0%)	
Other	4,584 (7.4%)	0 (0.0%)	
Diabetes	**12,672 (24.7%)**	**171 (15.6%)**	**<0.001**
History of prior cardiac surgery	**13,838 (22.5%)**	**159 (13.7%)**	**<0.001**
Donor age, mean (SD)	**31.258 (11.772)**	**32.967 (12.637)**	**<0.001**
Ischemic time, mean (SD), hours	**3.085 (1.064)**	**3.245 (1.084)**	**<0.001**
Use of intra-aortic balloon pump	**5,620 (9.1%)**	**128 (11.1%)**	**0.024**
Ventricular assist device	**17,227 (28.0%)**	**101 (8.7%)**	**<0.001**
Ventilator use at transplant	1,340 (2.2%)	17 (1.5%)	0.101
Days waited for transplant, mean (SD)	**208.326 (341.108)**	**145.125 (252.631)**	**<0.001**
Serum creatinine, mean (SD), mg/dl	**1.293 (0.884)**	**1.302 (0.506)**	**0.016**
PA systolic pressure, mean (SD), mm Hg	**41.666 (14.532)**	**42.423 (12.480)**	**0.005**
PA diastolic pressure, mean (SD), mm Hg	**20.176 (8.840)**	**20.757 (7.353)**	**<0.001**
PA mean pressure, mean (SD), mm Hg	**28.227 (10.387)**	**29.076 (8.632)**	**<0.001**
PCWP, mean (SD), mm Hg	**18.786 (9.022)**	**19.906 (7.430)**	**<0.001**
Cardiac output, mean (SD), liter/min	**4.493 (1.485)**	**4.073 (1.329)**	**<0.001**
Most recent listing status, no. (%)			
Status 1A	**18,202 (30.3%)**	**391 (34.1%)**	**<0.001**
Status 1B	12,505 (20.8%)	255 (22.2%)	
Status 2	10,589 (17.6%)	144 (12.5%)	
Old status 1	9,231 (15.4%)	73 (6.4%)	
Status 1	779 (1.3%)	12 (1.0%)	
Status 2	4,205 (7.0%)	145 (12.6%)	
Status 3	1,644 (2.7%)	48 (4.2%)	
Status 4	1,746 (2.9%)	73 (6.4%)	
Status 5	3 (0.0%)	0 (0.0%)	
Status 6	466 (0.8%)	7 (0.6%)	
Status 7	764 (1.3%)	0 (0.0%)	
History of smoking	**23,765 (38.6%)**	**321 (27.7%)**	**<0.001**
History of dialysis	975 (1.6%)	21 (1.8%)	0.533
Panel reactive antibodies (%)	10.434 (22.093)	9.447 (20.458)	0.164
No. listed by era			
1987-2000	**20,770 (33.7%)**	**162 (14.0%)**	**<0.001**
2001-2013	20,953 (34.0%)	406 (35.1%)	
2014-2022	19,829 (32.2%)	589 (50.9%)	
Cause of death, no. (%)			
Non-CV	**23,424 (71.2%)**	**346 (78.5%)**	**<0.001**
CV	9,481 (28.8%)	95 (21.5%)	

Abbreviations: PA, pulmonary artery; PCWP, Pulmonary Capillary Wedge Pressure; CV, Cardiovascular.

Kruskal-Wallis test for continuous variables. Pearson's test for categorical variables.

The bold is to help highlight any statistically signficant results with p<0.05.

### RCM and non-RCM cohorts

The clinical and demographic characteristics of the RCM patients are shown in Table 1. Compared to the non-RCM cohort, RCM patients were more likely to be older (54.7±13.3 vs 52.7±11.9, *p* < 0.001), female (34.7% vs 23.9%, *p* < 0.001), and Black (21% vs 17%, *p* < 0.001). RCM patients also had lower incidence of diabetes (15.6% vs 24.7%, *p*<0.001), history of non-transplant cardiac surgery (13.7% vs 22.5%, *p* < 0.001), and smoking (27.7% vs 38.6%, *p* < 0.001). There were no differences in history of dialysis or PRA.

Donor age was significantly higher (33±12.7 vs 31.3± 11.8, *p*<0.001) with longer ischemic times (3.25 vs. 3.09 hours, *p*<0.001) in the RCM group. Additionally, the RCM group had lower incidence of ventricular assist devices use (8.7% vs 28%, *p*<0.001), but higher utilization of intra-aortic balloon pumps (11.1% vs 9.1%, *p* < 0.001). The RCM cohort also had a significantly shorter time on the waitlist (145 vs 208 days, *p* < 0.001) and higher status on the waitlist in both the pre-2018 (34.1% vs 30.3% status 1A, *p* = 0.024) and post-2018 (12.6% vs 7% status 2, *p*<0.001). The RCM cohort had significantly increased pulmonary artery pressures as well as pulmonary capillary wedge pressure (7.4 vs 9 mm Hg, *p* < 0.001) and cardiac output (4.07 vs 4.5 liter/min, *p* < 0.001). The RCM cohort was less likely to die from cardiovascular death compared to the non-RCM group (21.5% vs 28.8%, *p* < 0.001).

### RCM subtype analysis

Subtype analysis of the RCM patients demonstrated a predominance of amyloid (49.4%) and idiopathic (31.2%) patients compared to radiation/chemotherapy (8.4%) and other (11%) (Table 2). Amyloid patients were significantly older than all other subtypes (61.7 vs 47-49.7 years, *p* < 0.001), male (80.2%), with worse renal function (Cr 1.34 *p* < 0.001. Chemotherapy/radiation patients were more likely to be females (66%, *p* < 0.001), diabetic (26% vs 13.4-16.9%, *p* ≤ 0.014), have history of cardiac surgery (45.4% vs 7.2-22.8% *p* < 0.001) with longer waitlist times (223 vs 100-186 days, *p* < 0.001) Traditional cardiac risk factors, such as diabetes, smoking, and dialysis, were relatively uncommon across all subgroups aside from smoking in idiopathic RCM (30.2% vs 24.4-27.3%, *p* < 0.001) There were no significant differences in dialysis, ventilator use, PRA, and cardiac death. Time on the waitlist was the shortest for amyloid patients (100 days), followed by idiopathic (180 days), other (186 days), and radiation/chemotherapy (223 days) (*p* < 0.001). Listing prevalence continuously increased by era in amyloid (6.5%/29.9%/63.6%) and chemotherapy/radiation (8.2%/42.3%/49.5%) in the *classic/contemporary/current* era analyses (*p* < 0.001) (Table S1).

**Table 2:** Baseline Characteristics and Clinical Data for RCM Subtypes Heart-Only Transplant Recipients

Variable	Idiopathic	Amyloid	Radiation/chemotherapy	Other	*p*-value
N	361 (31.2%)	572 (49.4%)	97 (8.4%)	127 (11.0%)	
Recipient age, mean (SD), years	47.019 (13.934)	61.722 (8.420)	49.670 (11.201)	48.646 (13.849)	**<0.001**
Recipient sex					
Female	**155 (42.9%)**	**113 (19.8%)**	**64 (66.0%)**	**69 (54.3%)**	**<0.001**
Male	206 (57.1%)	459 (80.2%)	33 (34.0%)	58 (45.7%)	
Ethnicity					
White	**280 (77.6%)**	**349 (61.0%)**	**76 (78.4%)**	**106 (83.5%)**	**<0.001**
Black	43 (11.9%)	179 (31.3%)	14 (14.4%)	7 (5.5%)	
Hispanic	27 (7.5%)	27 (4.7%)	5 (5.2%)	7 (5.5%)	
Asian	9 (2.5%)	14 (2.4%)	2 (2.1%)	6 (4.7%)	
American Indian/Alaska Native	0 (0.0%)	1 (0.2%)	0 (0.0%)	0 (0.0%)	
Native Hawaiian/Other Pacific Islander	2 (0.6%)	2 (0.3%)	0 (0.0%)	0 (0.0%)	
Multiracial	0 (0.0%)	0 (0.0%)	0 (0.0%)	1 (0.8%)	
Diabetes	**55 (16.9%)**	**75 (13.5%)**	**25 (26.0%)**	**16 (13.4%)**	**0.014**
History of prior cardiac surgery	**45 (12.5%)**	**41 (7.2%)**	**44 (45.4%)**	**29 (22.8%)**	**<0.001**
Donor age, mean (SD)	**31.615 (12.607)**	**34.507 (12.708)**	**32.856 (12.655)**	**29.961 (11.491)**	**<0.001**
Ischemic time, mean (SD), hours	3.230 (1.080)	3.221 (1.027)	3.467 (1.340)	3.234 (1.131)	0.447
Intra-aortic balloon pump	**27 (7.5%)**	**89 (15.6%)**	**4 (4.1%)**	**8 (6.3%)**	**<0.001**
Ventricular assist device	**46 (12.7%)**	**32 (5.6%)**	**15 (15.5%)**	**8 (6.3%)**	**<0.001**
Ventilator use at transplant	8 (2.2%)	6 (1.0%)	1 (1.0%)	2 (1.6%)	0.527
Days waited for transplant, mean (SD)	180.127 (316.138)	100.659 (156.555)	223.423 (362.176)	186.102 (264.734)	**<0.001**
Serum creatinine, mean (SD) mg/dl	**1.279 (0.580)**	**1.338 (0.469)**	**1.273 (0.480)**	**1.222 (0.466)**	**<0.001**
PA systolic pressure, mean (SD), mm Hg	43.010 (13.680)	42.767 (11.428)	39.340 (13.052)	41.748 (13.162)	0.052
PA diastolic pressure, mean (SD), mm Hg	**21.828 (8.485)**	**20.670 (6.717)**	**18.979 (7.421)**	**19.757 (6.490)**	**0.009**
PA mean pressure, mean (SD), mm Hg	29.795 (9.753)	29.087 (7.757)	27.489 (9.307)	28.390 (8.711)	0.079
PCWP, mean (SD), mm Hg	**20.578 (8.126)**	**20.111 (6.797)**	**18.293 (8.396)**	**18.522 (7.200)**	**0.013**
Cardiac output, mean (SD), liter/min	4.169 (1.412)	3.963 (1.167)	4.481 (1.836)	4.004 (1.268)	0.054
Most recent listing status					
Status 1A	115 (32.4%)	199 (34.9%)	35 (36.1%)	42 (33.6%)	**<0.001**
Status 1B	86 (24.2%)	116 (20.3%)	26 (26.8%)	27 (21.6%)	
Status 2	63 (17.7%)	47 (8.2%)	12 (12.4%)	22 (17.6%)	
Old status 1	46 (13.0%)	17 (3.0%)	3 (3.1%)	7 (5.6%)	
Status 1	4 (1.1%)	6 (1.1%)	1 (1.0%)	1 (0.8%)	
Status 2	17 (4.8%)	104 (18.2%)	10 (10.3%)	14 (11.2%)	
Status 3	10 (2.8%)	33 (5.8%)	3 (3.1%)	2 (1.6%)	
Status 4	13 (3.7%)	46 (8.1%)	4 (4.1%)	10 (8.0%)	
Status 6	1 (0.3%)	3 (0.5%)	3 (3.1%)	0 (0.0%)	
History of smoking	109 (30.2%)	156 (27.3%)	25 (25.8%)	31 (24.4%)	0.565
History of dialysis	7 (1.9%)	8 (1.4%)	3 (3.1%)	3 (2.4%)	0.639
Panel reactive antibodies (%)	9.209 (20.516)	8.865 (20.412)	10.647 (21.818)	12.103 (19.723)	0.123
No. listed by era					
1987-2000	**98 (27.1%)**	**37 (6.5%)**	**8 (8.2%)**	**19 (15.0%)**	**<0.001**
2001-2013	**146 (40.4%)**	**171 (29.9%)**	**41 (42.3%)**	**48 (37.8%)**	
2014-2022	**117 (32.4%)**	**364 (63.6%)**	**48 (49.5%)**	**60 (47.2%)**	
Cause of death					
Non-CV	129 (75.0%)	149 (81.0%)	40 (85.1%)	28 (73.7%)	0.302
CV	43 (25.0%)	35 (19.0%)	7 (14.9%)	10 (26.3%)	

Abbreviations: PA, pulmonary artery. PCWP, pulmonary capillary wedge pressure; CV, Cardiovascular.

Kruskal-Wallis test for continuous variables. Pearson's test for categorical variables.

The bold is to help highlight any statistically signficant results with p<0.05.

### Comparison of different transplant eras

Comparison of the *classic* (1987-2000) *contemporary* (2001-2013), and *current* (2014-2022) eras demonstrated interesting temporal changes. Age of both donors and recipients increased across the eras (28.9/47.9 years, 32.4/52.0 years, and 34.5/58.4 years, respectively (*p* < 0.001) (Table S1). Ischemic time also increased significantly (2.99 vs 3.29 hours, *classic* vs *current*, *p* = 0.011) while utilization of intra-aortic balloon pumps increased (1.9% vs 3.9% vs 18.5%, *p* < 0.001). Patients had progressively higher listing status with each progressive era (5.2% status 1A *classic* vs 44.8% *contemporary*) with significantly decreased waitlist times (167 vs 145 vs 139 days, *p* = 0.002). Interestingly, the incidence of PRAs was significantly higher in the *contemporary* era (25.8%).

### Survival, waitlist, and competing outcomes

#### Post-transplant

**Figure 1:**
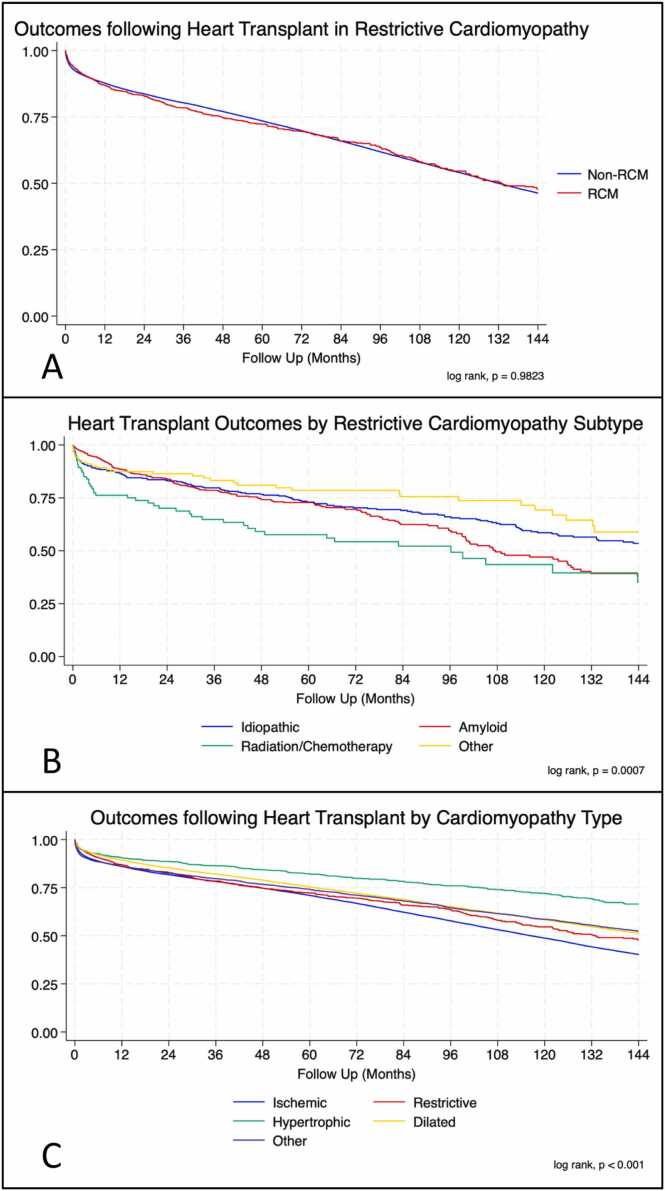
Kaplan Meier curves for restrictive and nonrestrictive cardiomyopathy (A), restrictive subtypes (B), and the other common cardiomyopathies (C).

**Figure 2:**
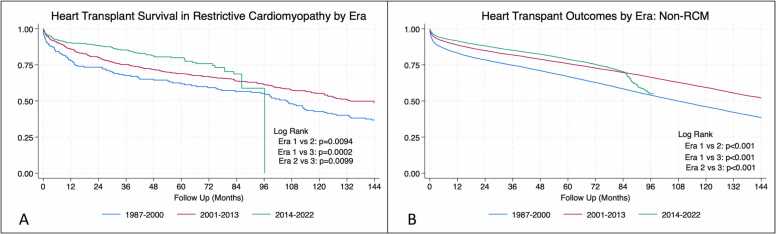
Survival outcomes of restrictive (A) and nonrestrictive (B) cardiomyopathy in the *classic* (1987-2000), *contemporary* (2001-2013), and *current* (2014-present) eras.

**Figure 3:**
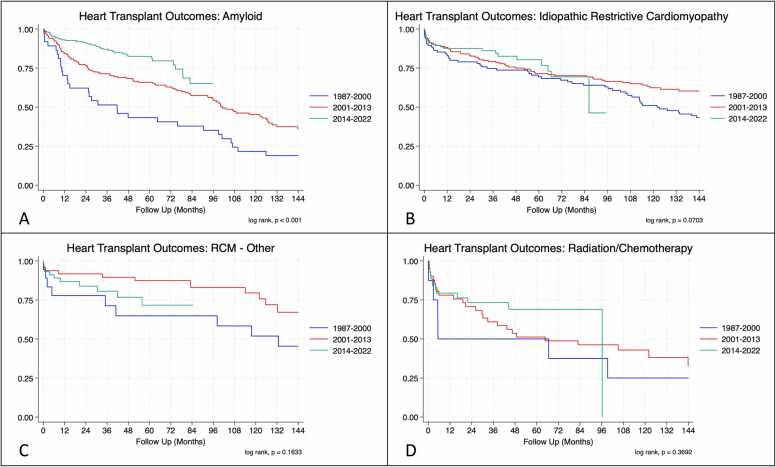
Survival curves for amyloid (A), idiopathic (B), other (C), and radiation/chemotherapy (D) restrictive cardiomyopathies stratified by *classic* (1987-2000), *contemporary* (2001-2013), and *current* (2014-2022) eras.

There was no significant difference in heart transplant survival between RCM and non-RCM recipients for the overall population (log rank, *p* = 0.98) (Figure 1A). When comparing RCM subtypes, radiation/chemotherapy patients fared significantly worse than all other RCM subtypes throughout the assessment period (log rank, *p* = 0.0007) (Figure 1B). Notably, while short-term amyloid survival has improved there appears to be worse long-term survival. Temporal analysis showed significant improvements in survival of all 4 subtypes with each progressive era. Looking at the entire non-RCM cohort short and medium-term survival at 1 and 5 years was significantly improved in each progressive era *current* (79%) > *contemporary* (59%) > *classic* (46%) (*p* < 0.001 (Figure 2B). These findings were mirrored in the RCM cohort with significantly improved survivorship in the *current* era (80%) as compared with the *classic* (43%) and *contemporary* eras (55%) (*p* < 0.001 and *p* = 0.009, respectively) (Figure 2A). Individualized subgroup survival analyses showed striking improvements in the amyloid and radiation/chemotherapy groups with each progressive era at 10 years: amyloid (22%/45%/83%) and radiation/chemotherapy (25%/43%/69%) (Figure 3).

Cox proportional hazards model for interval-censored survival-time data showed notable findings. At baseline, RCM patients had worse survival compared to all other cardiomyopathies except ischemic cardiomyopathy Hazard Ratio (HR) 1.16 (95% Cardiovascular Confidence Interval (CI): 1.05-1.29, *p* = 0.004) (Table S3). Temporal assessment showed the *current* (2014-2022) era has the best survival as compared to the *classic* (1987-2000) (HR 1.98 (95% CI: 1.47-2.66, *p* < 0.001) RCM; HR1.72 (95% CI:1.65-1.79, *p* < 0.001) non-RCM) and *contemporary* (2001-2013) (HR 1.43 (95% CI:1.10-1.86, *p* = 0.007) RCM; HR 1.16 (95% CI:1.12-1.21, *p* < 0.0001) non-RCM) eras (Table S5). Subtype analysis demonstrated that chemotherapy/radiation patients had the worst overall prognosis HR 1.68 (95% CI: 1.23-2.31, *p* = 0.001). Compared to amyloid, chemotherapy/radiation patients held a higher risk HR 1.48 (95% CI: 1.06-2.06, *p* = 0.020) while a diagnosis of “other” was protective HR 0.65 (95% CI: 0.45-0.95, *p* = 0.026) (Table S4). On par with the survival trends, amyloid outcomes appear to have peaked in the *current* era as compared to *classic* HR 3.55 (95% CI: 2.22-5.70, *p* < 0.001) and *contemporary* HR 1.99 (95% CI: 1.37-2.88, *p* < 0.001) eras.

### Waiting list

**Figure 4:**
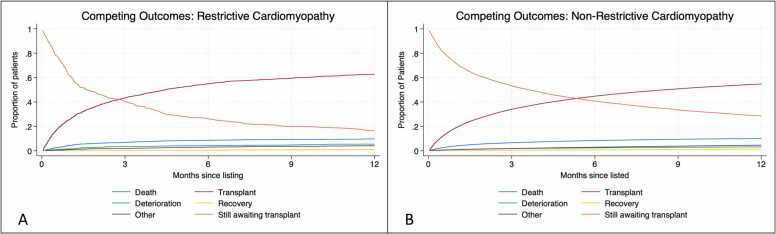
Competing outcomes analysis of waitlist patients with restrictive (A) and nonrestrictive (B) cardiomyopathy.

Analysis of the cohort awaiting transplant included 97,790 non-RCM and 1,695 RCM patients. In comparing these groups, the RCM patients were significantly older 53.0 vs 51.7 years (*p* < 0.001), female (34.7% vs 23.8%, *p* < 0.001), and had a low incidence of smoking (28.3% vs 39.1%, *p* < 0.001) and diabetes (14.4% vs 25%, *p* < 0.001) (Table S2). Other notable findings were that these patients were presumably less sick, with significantly lower utilization of ventricular assist devices or mechanical ventilation (*p* < 0.0001). Competing outcomes analysis also showed unique differences in both the RCM and non-RCM cohorts. The transplant rate was much higher in the RCM compared to non-RCM patients at 3, 6, and 12 months but it was accompanied with a higher deterioration and death rate (Figure 4). Recovery was less common in the RCM cohort (1.02% vs 1.47% at 12 months) (Table S2).

## Discussion

RCM encompasses a wide variety of disorders with distinct etiologies that all lead to progressive heart failure and high mortality if not treated. Historically, diagnostic strategies and targeted therapies for RCM were very limited and patients were often diagnosed late in their disease course, leading to a very poor prognosis. However, with heightened awareness of amyloidosis and RCM in the medical community, the advent of improved diagnostic and therapeutic modalities such as pyrophosphate scan/protein stabilizers for amyloid and expanded genetic assays familial RCM the overall survival appears to be improving.^10^, ^11^, ^12^ Ultimately, heart transplantation remains the gold standard for long-term survival, but data from large studies are lacking, mainly limited to small case series and single-center studies.^5^ As such we sought to describe contemporary incidence, trends, and outcomes of RCM transplantation in the United States.

The prevalence of RCM was rare (1.7%); however, this proportion has increased with time.^9^ The increasing prevalence is likely multifactorial including improved medical therapies for common disorders, such as cancer, improved strategies for amyloid as well as improved diagnostic testing for RCM subtype identification. Subtype analysis of the RCM patients demonstrated a higher incidence of amyloid (49.4%) and idiopathic (31.2%) patients compared to radiation/chemotherapy (8.4%) and other (11%). The higher prevalence of amyloid be attributable to an aging US population and more widespread availability of Cardiac Magnetic Resonance Imaging/Positron emission tomography/pyrophosphate scans. Temporal analysis showed that transplant centers are being more aggressive with both donor age, recipient age, and ischemic times and as such have significantly improved transplantation rates and shortened waitlist times which in accordance with other recent analyses.^6^, ^13^ Whether this will portend worse long-term outcomes is to be seen, but our most recent dataset from the *current* era demonstrates promising results for both waiting list outcomes and overall survival.

Overall post heart transplant survival in RCM patients demonstrated no difference to non-RCM patients. However, analysis of the *classic, contemporary*, and *current* transplant eras demonstrated significant improvements in both pre, post, and long-term transplant management as shown by overall survival and improved hazard ratios. These findings are likely multifactorial including the UNOS allocation change, improved surveillance and rejection treatment strategies, and greater awareness of RCM physiology with earlier diagnosis.^14^, ^15^

Subgroup analyses also highlighted some very important findings. RCM patients had a significantly lower incidence of traditional cardiac risk factors compared to non-RCM patients which may explain some of the improved outcomes. The RCM cohort had significantly lower utilization of left ventricular assist devices which is likely secondary to RCM physiology waitlist times improved as well, particularly for amyloid patients, which may be attributable to improved recognition, changes in UNOS allocation and use of exceptions for higher listing status. Heart transplant outcomes in recipients with RCM secondary to chemotherapy/radiation continue to have worse outcomes with high post-operative mortality.^16^ However, the survival in this subgroup has improved in the more recent era. This may be secondary to earlier recognition, improved pretransplant management strategies, and changes in UNOS allocation in this subtype.

Detailed clinical and echocardiographic information including amyloid subtype analysis is not available in the Heart Transplant Candidate Registration forms within the UNOS registry. As a consequence, we are unable to distinguish between Amyloid Light Chain and Transthyretin amyloid as well as confirm the RCM diagnosis. Additionally, radiation/chemotherapy is listed as a singular diagnosis and it is not possible to analyze separately. Multiorgan recipients were also excluded.

## Conclusions

Heart transplantation due to RCM represents a smaller proportion of overall volume; however, prevalence has increased in the more recent era. Overall outcomes following heart transplantation are comparable to non-RCM recipients and improved in the more recent eras despite more aggressive donor and recipient selection coupled with changes in UNOS allocation. Amyloid, the most common RCM subtype, demonstrated improved post-transplant survival by era. However, long-term survival was diminished and may be attributable to the systemic effects of amyloid. Additionally, recipients with RCM secondary to chemotherapy/radiation demonstrated the worst survival amongst the RCM subtypes. This has improved in the more recent era but further study is warranted to assess mitigating factors.

Further research is needed on the incidence, predictive factors, and diagnostic/therapeutic modalities available for identification and successful transplantation of RCM in all subtypes. Future analysis would benefit from incorporation of more granular data on subtypes of RCM, with special focus upon at risk populations such as chemotherapy/radiation and amyloid RCM.

## Author Contributions

D.M. was responsible for data interpretation and manuscript preparation. E.D. assisted with data collection, statistical analysis, and manuscript editing.
