# Supplementary material for: Heart transplant outcomes in restrictive cardiomyopathy: UNOS registry analysis of the last three decades
Source: JHLT Open. 2023 Dec 5;3:100031. doi: 10.1016/j.jhlto.2023.100031 (PMC11935335; doi:10.1016/j.jhlto.2023.100031)
Supplement: Supplementary file 1 — Supplementary material [file mmc1.docx]

**Supplementary Figure 1: Heart-Liver Transplantation Trends in Amyloid Patients**


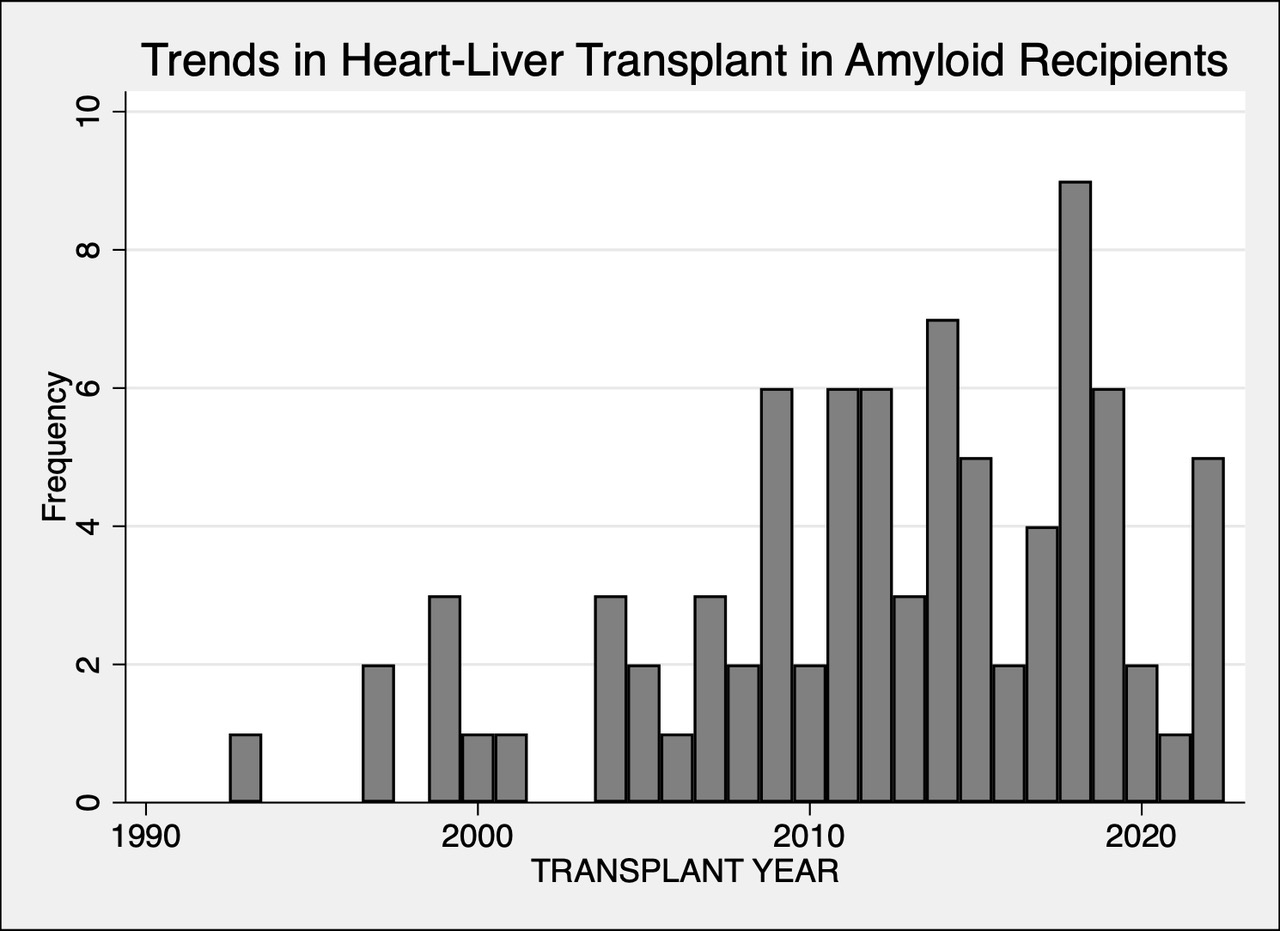


| **Supplementary Table 6A. Heart-Kidney and Heart-Liver Transplantation for RCM Subtypes for Heart-Only Transplant Recipients** | | | | | |  |
| --- | --- | --- | --- | --- | --- | --- |
|  | **Idiopathic** | **Amyloid** | **Radiation/Chemotherapy** | **Other** | **p-value** | |
| Total (%) | 439 (28.8%) | 803 (52.8%) | 118 (7.8%) | 162 (10.6%) |  | |
| Heart-Kidney |  |  |  |  |  | |
| No | 424 (96.6%) | 729 (90.8%) | 107 (90.7%) | 155 (95.7%) | <0.001 | |
| Yes | 15 (3.4%) | 74 (9.2%) | 11 (9.3%) | 7 (4.3%) |  | |
| Heart-Liver |  |  |  |  |  | |
| No | 431 (98.2%) | 720 (89.7%) | 117 (99.2%) | 154 (95.1%) | <0.001 | |
| Yes | 8 (1.8%) | 83 (10.3%) | 1 (0.8%) | 8 (4.9%) |  | |
| Pearson's test for categorical variables. | | | | | |  |

| **Supplementary Table 6B. Heart-Kidney and Heart-Liver Transplantation for RCM and Non-RCM Heart-Only Transplant Recipients** | | | |
| --- | --- | --- | --- |
|  | **Non-RCM** | **RCM** | **p-value** |
| Total (%) | 73,346 (98.0%) | 1,522 (2.0%) |  |
| Heart-Kidney |  |  |  |
| No | 70,786 (96.5%) | 1,415 (93.0%) | <0.001 |
| Yes | 2,560 (3.5%) | 107 (7.0%) |  |
| Heart-Liver |  |  |  |
| No | 72,927 (99.4%) | 1,422 (93.4%) | <0.001 |
| Yes | 419 (0.6%) | 100 (6.6%) |  |
| Pearson's test for categorical variables. | | | |
