# Supplementary material for: Heart transplant outcomes in restrictive cardiomyopathy: UNOS registry analysis of the last three decades
Source: JHLT Open. 2023 Dec 5;3:100031. doi: 10.1016/j.jhlto.2023.100031 (PMC11935335; doi:10.1016/j.jhlto.2023.100031)
Supplement: Supplementary file 2 — Supplementary material [file mmc2.docx]

| **Supplementary Table 1. Baseline Characteristics and Clinical Data for RCM Heart-Only Transplant Recipients by Era** | | | | |
| --- | --- | --- | --- | --- |
|  | ***Classic*** | ***Contemporary*** | ***Current*** | **p-value** |
|  | **1987-2000** | **2001-2013** | **2014-2022** |  |
| N | 162 (14.0%) | 406 (35.1%) | 589 (50.9%) |  |
| Recipient Age | **47.883 (11.681)** | **51.983 (13.711)** | **58.426 (12.121)** | **<0.001** |
| Recipient Sex |  |  |  |  |
| Female | **72 (44.4%)** | **173 (42.6%)** | **156 (26.5%)** | **<0.001** |
| Male | 90 (55.6%) | 233 (57.4%) | 433 (73.5%) |  |
| Ethnicity |  |  |  |  |
| White | **139 (85.8%)** | **312 (76.8%)** | **360 (61.1%)** | **<0.001** |
| Black | 15 (9.3%) | 59 (14.5%) | 169 (28.7%) |  |
| Hispanic | 4 (2.5%) | 22 (5.4%) | 40 (6.8%) |  |
| Asian | 4 (2.5%) | 12 (3.0%) | 15 (2.5%) |  |
| American Indian/Alaska Native | 0 (0.0%) | 0 (0.0%) | 1 (0.2%) |  |
| Native Hawaiian/Other Pacific Islander | 0 (0.0%) | 1 (0.2%) | 3 (0.5%) |  |
| Multiracial | 0 (0.0%) | 0 (0.0%) | 1 (0.2%) |  |
| Diabetes | **5 (4.9%)** | **48 (11.8%)** | **118 (20.0%)** | **<0.001** |
| History of Prior Cardiac Surgery | **0 (0.0%)** | **72 (17.7%)** | **87 (14.8%)** | **<0.001** |
| Donor Age, Mean (SD) | **28.877 (13.198)** | **32.367 (13.166)** | **34.506 (11.816)** | **<0.001** |
| Ischemic Time, Mean (SD), hrs | **2.997 (0.935)** | **3.281 (1.090)** | **3.286 (1.109)** | **0.011** |
| Intra-aortic Balloon Pump | **3 (1.9%)** | **16 (3.9%)** | **109 (18.5%)** | **<0.001** |
| Ventricular Assist Device | 7 (4.3%) | 40 (9.9%) | 54 (9.2%) | 0.094 |
| Ventilator Use at Transplant | 5 (3.1%) | 7 (1.7%) | 5 (0.8%) | 0.097 |
| Days waited for transplant, Mean (SD) | **167.173 (226.202)** | **144.665 (264.593)** | **139.379 (251.149)** | **0.002** |
| Serum Creatinine, Mean (SD), mg/dL | 1.438 (0.725) | 1.288 (0.531) | 1.288 (0.434) | 0.129 |
| PA systolic pressure, Mean (SD), mmHg | **46.485 (15.600)** | **41.869 (12.117)** | **42.110 (12.025)** | **0.040** |
| PA diastolic pressure, Mean (SD), mmHg | **23.959 (9.311)** | **20.755 (7.355)** | **20.226 (6.846)** | **<0.001** |
| PAP mean pressure, Mean (SD), mmHg | **32.274 (10.995)** | **28.910 (8.484)** | **28.657 (8.184)** | **0.015** |
| PCWP, Mean (SD), mmHg | **23.041 (9.117)** | **19.836 (7.128)** | **19.404 (7.176)** | **0.002** |
| Cardiac output, Mean (SD), L/min | 4.027 (1.426) | 4.059 (1.436) | 4.090 (1.241) | 0.515 |
| Most Recent Listing Status, No. (%) |  |  |  |  |
| * Status 1A | **8 (5.2%)** | **182 (44.8%)** | **201 (34.1%)** | **<0.001** |
| * Status 1B | 19 (12.4%) | 153 (37.7%) | 83 (14.1%) |  |
| * Status 2 | 53 (34.6%) | 71 (17.5%) | 20 (3.4%) |  |
| ** Old Status 1 | 73 (47.7%) | 0 (0.0%) | 0 (0.0%) |  |
| Status 1 | 0 (0.0%) | 0 (0.0%) | 12 (2.0%) |  |
| Status 2 | 0 (0.0%) | 0 (0.0%) | 145 (24.6%) |  |
| Status 3 | 0 (0.0%) | 0 (0.0%) | 48 (8.1%) |  |
| Status 4 | 0 (0.0%) | 0 (0.0%) | 73 (12.4%) |  |
| Status 6 | 0 (0.0%) | 0 (0.0%) | 7 (1.2%) |  |
| History of Smoking | 24 (14.8%) | 111 (27.3%) | 186 (31.6%) | **<0.001** |
| History of Dialysis | 0 (0.0%) | 10 (2.5%) | 11 (1.9%) | 0.138 |
| Panel Reactive Antibodies (%) | **3.149 (11.776)** | **17.447 (25.799)** | **8.325 (19.336)** | **<0.001** |
| Cause of Death |  |  |  |  |
| Non-CV | 105 (77.2%) | 165 (78.9%) | 76 (79.2%) | 0.912 |
| CV | 31 (22.8%) | 44 (21.1%) | 20 (20.8%) |  |
| Kruskal-Wallis test for continuous variables. Pearson's test for categorical variables. | | | | |

**Supplementary Table 2. Competing Outcomes Analysis of Restrictive and Non-Restrictive Cardiomyopathies**

| **RCM: 3 Month Outcomes** | | | | | |
| --- | --- | --- | --- | --- | --- |
| **Variable** | **Observed** | **Mean** | **Standard Deviation** | **Min** | **Max** |
| Death | 5 | 6.89% | 0.0008023 | 0.0681013 | 0.0698953 |
| Transplant | 22 | 43.49% | 0.0037244 | 0.4300642 | 0.44023 |
| Deterioration | 1 | 3.44% | 0.00 | 0.034388 | 0.034388 |
| Recovery | 0 | 0% | 0.00 | 0.00 | 0.00 |
| Other | 3 | 1.84% | 0.000598 | 0.0177786 | 0.0189745 |
| Waiting | 4 | 40.34% | 0.0064861 | 0.3969072 | 0.4123711 |
| **RCM: 6 Month Outcomes** | | | | | |
| **Variable** | **Observed** | **Mean** | **Standard Deviation** | **Min** | **Max** |
| Death | 1 | 8.55% | 0.00 | 0.0854785 | 0.0854785 |
| Transplant | 11 | 54.97% | 0.001857 | 0.5475647 | 0.5523917 |
| Deterioration | 1 | 4.28% | 0.00 | 0.0427903 | 0.0427903 |
| Recovery | 1 | 0.48% | 0.00 | 0.0047757 | 0.0047757 |
| Other | 2 | 2.95% | 0.0004266 | 0.0291715 | 0.0297748 |
| Waiting | 1 | 26.29% | 0.00 | 0.2628866 | 0.2628866 |
|  |  |  |  |  |  |
| **RCM: 12 Month Outcomes** | | | | | |
| **Variable** | **Observed** | **Mean** | **Standard Deviation** | **Min** | **Max** |
| Death | 32 | 9.70% | 0.00 | 0.0969884 | 0.0969884 |
| Transplant | 128 | 62.81% | 0.0000538 | 0.6274884 | 0.6280967 |
| Deterioration | 20 | 5.43% | 0.00 | 0.0542983 | 0.0542983 |
| Recovery | 44 | 1.02% | 0.00 | 0.0102284 | 0.0102284 |
| Other | 42 | 4.07% | 0.00 | 0.0406818 | 0.0406818 |
| Waiting | 32 | 16.49% | 0.00 | 0.1649485 | 0.1649485 |
| **Non-RCM: 3 Month Outcomes** | | | | | |
| **Variable** | **Observed** | **Mean** | **Standard Deviation** | **Min** | **Max** |
| Death | 142 | 5.19% | 0.0004105 | 0.0513844 | 0.0525085 |
| Transplant | 995 | 35.49% | 0.0028966 | 0.3507218 | 0.3591577 |
| Deterioration | 47 | 1.62% | 0.0001334 | 0.0159688 | 0.0163709 |
| Recovery | 17 | 0.44% | 0.0000461 | 0.0043879 | 0.0045116 |
| Other | 66 | 1.60% | 0.000193 | 0.0157351 | 0.0162817 |
| Waiting | 142 | 53.20% | 0.0037092 | 0.526512 | 0.5366695 |
| **Non-RCM: 6 Month Outcomes** | | | | | |
| **Variable** | **Observed** | **Mean** | **Standard Deviation** | **Min** | **Max** |
| Death | 57 | 6.59% | 0.0001739 | 0.0656599 | 0.0661477 |
| Transplant | 525 | 46.68% | 0.0015566 | 0.4645239 | 0.4690702 |
| Deterioration | 37 | 2.18% | 0.0001055 | 0.0216139 | 0.0219149 |
| Recovery | 18 | 0.75% | 0.0000544 | 0.0073562 | 0.0075223 |
| Other | 67 | 2.53% | 0.0001889 | 0.0249994 | 0.0255599 |
| Waiting | 56 | 40.69% | 0.0015329 | 0.4047153 | 0.409002 |
|  |  |  |  |  |  |
| **Non-RCM: 12 Month Outcomes** | | | | | |
| **Variable** | **Observed** | **Mean** | **Standard Deviation** | **Min** | **Max** |
| Death | 3,099 | 7.97% | 0.0000115 | 0.0795033 | 0.0797454 |
| Transplant | 10,918 | 57.11% | 0.0000669 | 0.5700947 | 0.5710838 |
| Deterioration | 1,599 | 2.90% | 0.00000263 | 0.0289753 | 0.029049 |
| Recovery | 3,805 | 1.47% | 0.00000422 | 0.0145361 | 0.0146623 |
| Other | 4,597 | 4.03% | 0.0000159 | 0.0400094 | 0.0403146 |
| Waiting | 3,083 | 28.49% | 0.0001024 | 0.2848756 | 0.2870189 |

**Supplementary Table 3. Cox Proportional Hazards Model for Interval-censored Survival-Time Data for Different Cardiomyopathies**

| **Cardiomyopathy** | **Hazard Ratio** | **Standard Error** | **z-value** | **p-value** | **[95% Confidence Interval]** | |
| --- | --- | --- | --- | --- | --- | --- |
| Ischemic | 1.163875 | .0606078 | 2.91 | 0.004 | 1.050947 | 1.288938 |
| Hypertrophic | .5811743 | .0467774 | -6.74 | 0.000 | 0.4963581 | 0.6804836 |
| Dilated | .879643 | .0459639 | -2.45 | 0.014 | 0.794015 | 0.9745052 |
| Other | .9069547 | .0518127 | -1.71 | 0.087 | 0.8108827 | 1.014409 |

**Supplementary Table 4. Cox Proportional Hazards Model for Interval-censored Survival-Time Data for Restrictive Cardiomyopathy Subtypes**

| **Overall** | | | | | | |
| --- | --- | --- | --- | --- | --- | --- |
| Cardiomyopathy | Hazard Ratio | Standard Error | z-value | p-value | [95% Confidence Interval] | |
| Amyloid | 1.156005 | 0.1211935 | 1.38 | 0.167 | .9412853 | 1.419705 |
| Chemotherapy/Radiation | 1.688327 | 0.2708581 | 3.26 | 0.001 | 1.232816 | 2.312143 |

| **Compared to Amyloid** | | | | | | | | | | |  |  |
| --- | --- | --- | --- | --- | --- | --- | --- | --- | --- | --- | --- | --- |
| Cardiomyopathy | Hazard Ratio | | Standard Error | | z-value | | p-value | [95% Confidence Interval] | | |  |  |
| Idiopathic | | 0.810492 | 0.0958311 | | -1.78 | | 0.076 | | | .6428419 | 1.021864 | |
| Chemotherapy/  Radiation | | 1.482018 | 0.2503049 | | 2.33 | | 0.020 | | | 1.064364 | 2.063559 | |
| Other | | 0.6500929 | 0.1255818 | | -2.23 | | 0.026 | | | .4451891 | 0.9493064 | |

**Supplementary Table 5. Cox Proportional Hazards Model for Interval-censored Survival-Time Data for Restrictive Cardiomyopathy Subtypes Compared to the *Current* [2014-20220] Era**

| **Restrictive Cardiomyopathy** | | | | | | | | | | |  |  |
| --- | --- | --- | --- | --- | --- | --- | --- | --- | --- | --- | --- | --- |
| Era | Hazard Ratio | | Standard Error | | z-value | | p-value | [95% Confidence Interval] | | |  |  |
| *Classic*  [1987-2000] | | 1.979479 | 0.2999865 | | 4.51 | | <0.001 | | | 1.470797 | 2.66409 | |
| *Contemporary* [2001-2013] | | 1.433488 | 0.1912229 | | 2.70 | | 0.007 | | | 1.103688 | 1.861837 |  |

| **Non-Restrictive Cardiomyopathy** | | | | | | | | | | |  |  |
| --- | --- | --- | --- | --- | --- | --- | --- | --- | --- | --- | --- | --- |
| Era | Hazard Ratio | | Standard Error | | z-value | | p-value | [95% Confidence Interval] | | |  |  |
| *Classic*  [1987-2000] | | 1.718955 | 0.0356145 | | 26.15 | | <0.001 | | | 1.65055 | 1.790195 | |
| *Contemporary* [2001-2013] | | 1.163449 | 0.0249243 | | 7.07 | | <0.001 | | | 1.11561 | 1.21334 |  |

| **Amyloid** | | | | | | | | | | |  |
| --- | --- | --- | --- | --- | --- | --- | --- | --- | --- | --- | --- |
| Era | Hazard Ratio | | Standard Error | | z-value | | p-value | [95% Confidence Interval] | | |  |
| *Classic*  [1987-2000] | | 3.552417 | 0.856247 | | 5.26 | | 0.000 | | | 2.214914 | 5.697587 |
| *Contemporary* [2001-2013] | | 1.985209 | 0.3753279 | | 3.63 | | 0.000 | | | 1.370491 | 2.875652 |

| **Chemotherapy/Radiation** | | | | | | | | | | |  |
| --- | --- | --- | --- | --- | --- | --- | --- | --- | --- | --- | --- |
| Era | Hazard Ratio | | Standard Error | | z-value | | p-value | [95% Confidence Interval] | | |  |
| *Classic*  [1987-2000] | | 1.961067 | 0.9882286 | | 1.34 | | 0.181 | | | 0.7303844 | 5.265423 |
| *Contemporary* [2001-2013] | | 1.424203 | 0.5019561 | | 1.00 | | 0.316 | | | 0.7137866 | 2.841681 |
